# Extranodal extension status is a powerful prognostic factor in stage III colorectal cancer

**DOI:** 10.18632/oncotarget.18223

**Published:** 2017-05-26

**Authors:** Chan Wook Kim, Jihun Kim, Seung-Seop Yeom, Jong Lyul Lee, Yong Sik Yoon, In Ja Park, Seok-Byung Lim, Seunghee Baek, Chang Sik Yu, Jin Cheon Kim

**Affiliations:** ^1^ Department of Surgery, University of Ulsan College of Medicine, Institute of Innovative Cancer Research, Asan Medical Center, Seoul, Korea; ^2^ Department of Pathology, University of Ulsan College of Medicine, Institute of Innovative Cancer Research, Asan Medical Center, Seoul, Korea; ^3^ Department of Clinical Epidemiology and Biostatistics, University of Ulsan College of Medicine, Asan Medical Center, Seoul, Korea

**Keywords:** colorectal cancer, lymph node, extranodal extension, stage III, prognosis

## Abstract

**Purpose:**

The present study aimed to evaluate the clinicopathologic characteristics of patients with extranodal extension (ENE) and the prognostic implications of ENE in stage III colorectal cancer (CRC).

**Results:**

ENE was more frequent in younger patients and those with rectal cancer, higher T stage, higher N stage, lymphovascular invasion (LVI), and perineural invasion (PNI). Five-year disease-free survival (DFS) and overall survival (OS) were lower in patients with ENE-positive than in those with ENE-negative tumors (DFS, 66.4% vs. 80.1%; and OS, 74.8% vs. 85.6%, respectively; *P* < 0.001 both). In multivariate analysis, pathologic stage, the presence of ENE, LVI, PNI, and no adjuvant chemotherapy were significant independent prognostic factors for DFS and OS. There were no statistically significant differences in DFS and OS between ENE-positive stage IIIB tumors and ENE-negative stage IIIC tumors.

**Materials and Methods:**

The records of 1,948 stage III CRC patients who underwent curative surgery between January 2003 and December 2010 were retrospectively reviewed.

**Conclusions:**

The presence of ENE is independently and significantly associated with lower DFS and OS rates after curative resection for stage III CRC. ENE status should be considered in both the pathologic report and CRC staging system.

## INTRODUCTION

Since 1987, the American Joint Committee on Cancer (AJCC) and the Union for International Cancer Control (UICC) have promoted a worldwide taxonomy of cancer staging based on the tumor-node-metastasis (TNM) staging system. Traditional TNM staging strategies for colorectal cancer (CRC) place patients with mesenteric lymph node involvement into the stage III category. A tumor nodule in the pericolonic fat without histologic evidence of residual lymph node tissue is classified as a tumor deposit. Previously, it was not considered a positive lymph node [1]. However, in the absence of unequivocal lymph node metastases, tumor deposits are considered N1 (particularly N1c) according to the AJCC 7^th^ edition [2].

Patients with stage III CRC are a heterogeneous group, and not all of these patients require strong adjuvant chemotherapy. Some of these patients have a good prognosis, similar to that of patients with stage II CRC, whereas others have a poor prognosis. In the AJCC staging system, the number of metastatic lymph nodes is the most important factor affecting prognosis in patients with stage III CRC. However, a variety of prognostic factors were identified that can be used to refine nodal staging, such as the ratio of the number of metastatic lymph nodes to retrieved lymph nodes [3–5], the presence of micrometastasis [6, 7], and the number of analyzed lymph nodes [8].

The presence of extranodal extension (ENE) of metastatic lymph nodes recently emerged as an important prognostic factor in several types of malignancies [9–15]. However, the prognostic value of ENE in CRC has not been reported extensively, and few studies are based on a reliable number of patients.

In the present study, we evaluated the clinicopathologic significance of ENE and investigated its prognostic implications in stage III CRC.

## RESULTS

### Patient characteristics

Of the 1,948 patients analyzed in this study, 1153 (59.2%) were male and 835 (42.9%) had rectal cancer. The mean age was 60 ± 11 years (range, 19–89 years). ENE in a metastatic lymph node was identified in 854 patients (43.8%). The mean follow-up interval was 62 ± 34 months.

### Clinicopathologic characteristics according to ENE status

There were no significant differences in sex, s-CEA level, and histologic type between ENE-positive and ENE-negative patients. However, ENE was more frequent in younger patients and those with rectal cancer, a higher T stage, a higher N stage, lymphovascular invasion (LVI), or perineural invasion (PNI) (Table 1).

**Table 1:** Clinicopathologic characteristics according to extranodal extension status

	Extranodal extension (-) (*n* = 1094)	Extranodal extension (+) (*n* = 854)	*P* value
**Sex**			0.747
Male	651 (59.5)	502 (58.8)	
Female	443 (40.5)	352 (41.2)	
**Age, years**			0.015
Mean ± SD	60 ± 11	59 ± 11	
**s-CEA level, ng/mL**			0.960
Mean ± SD	7.3 ± 31.9	7.4 ± 25.8	
**Tumor location**			0.003
Colon	657 (60.1)	456 (53.4)	
Rectum	437 (39.9)	398 (46.6)	
**pT category**			<0.001
T1	66 (5.9)	16 (1.9)	
T2	123 (11.2)	59 (6.9)	
T3	852 (77.9)	723 (84.7)	
T4	53 (4.8)	56 (6.6)	
**pN category**			<0.001
N1	901 (82.4)	456 (53.4)	
N2	193 (17.6)	398 (46.6)	
**pStage**			<0.001
IIIA	175 (16.0)	51 (6.0)	
IIIB	841 (76.9)	586 (68.6)	
IIIC	78 (7.1)	217 (25.4)	
**Histology**			0.191
WD/MD	992 (90.7)	759 (88.9)	
PD/SRC/Muc	102 (9.3)	95 (11.1)	
**LVI**	374 (34.2)	378 (44.3)	<0.001
**PNI**			<0.001
No	890 (81.4)	620 (72.6)	
Yes	183 (16.7)	219 (25.6)	
Unknown	21 (1.9)	15 (1.8)	
**Adjuvant CTx**			0.272
Yes	960 (87.8)	735 (86.1)	
No	134 (12.2)	119 (13.9)	
**Recurrence**	199 (18.2)	272 (31.9)	<0.001
**Follow-up months**			
Median (range)	64.8 (0.3–154.5)	60.8 (0.5–152.4)	

SD: standard deviation; s-CEA: serum carcinoembryonic antigen; WD: well-differentiated; MD: moderately differentiated; PD: poorly differentiated; SRC: signet ring cell; Muc: mucinous; LVI: lymphovascular invasion; PNI: perineural invasion; CTx: chemotherapy.

### Prognostic factors for DFS

**Figure 1:**
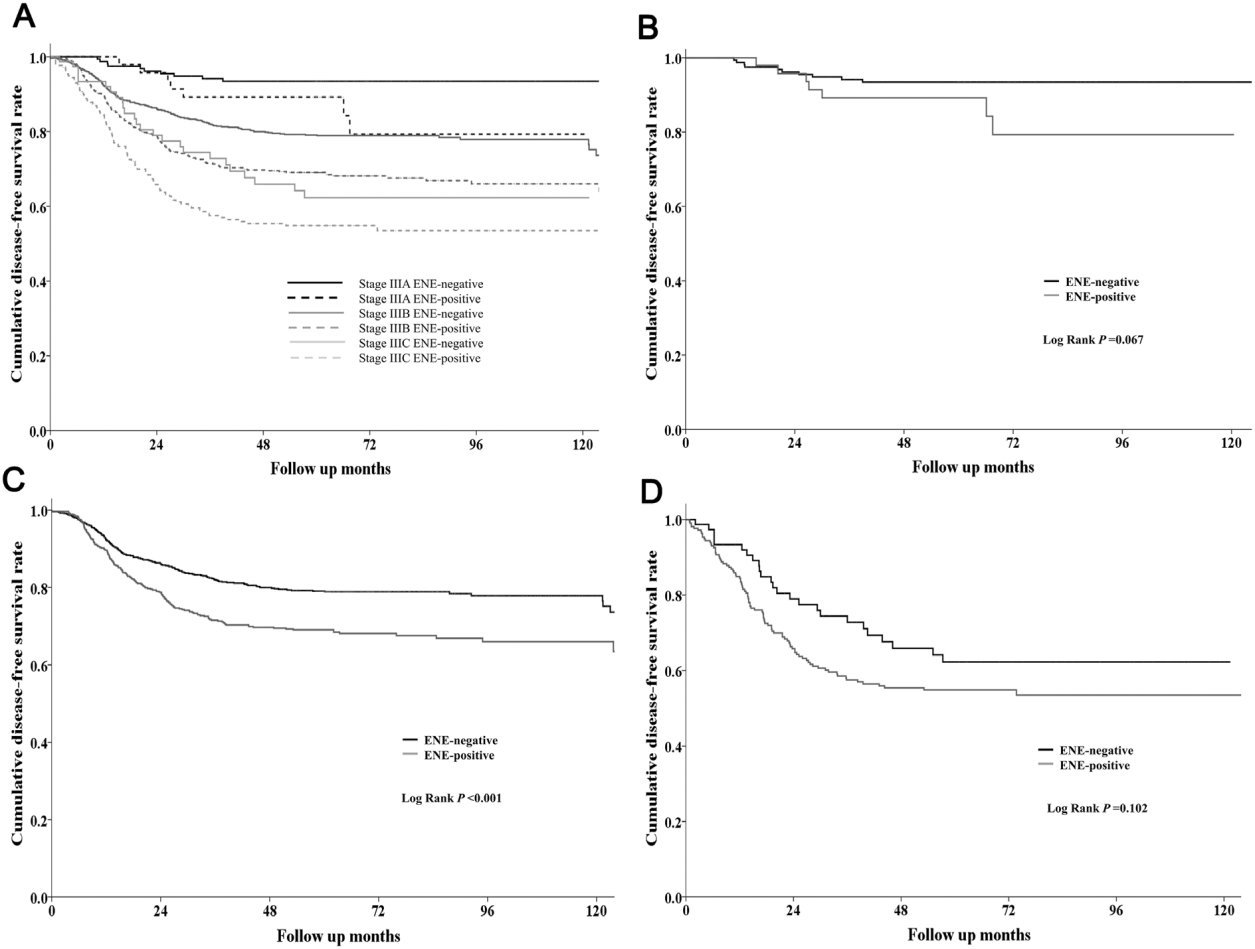
**(A)** Disease-free survival (DFS) rates according to the combination of stage and ENE status of stage III CRC patients. **(B)** DFS in stage IIIA patients with (*N* = 51) or without (*N* = 175) ENE. **(C)** DFS in stage IIIB patients with (*N* = 586) or without (*N* = 841) ENE. **(D)** DFS in stage IIIC patients with (*N* = 217) or without (*N* = 78) ENE.

The 5-year DFS rate was lower in patients with ENE-positive tumors than in patients with ENE-negative tumors (66.4% vs. 80.1%, *P* < 0.001). Comparison of DFS rates according to stage showed that the 5-year DFS rate was lower in the ENE-positive group than in the ENE-negative group in stage IIIB (66.0% vs. 80.1%, *P* < .001), whereas stage IIIA and IIIC patients showed similar DFS rates (ENE-positive vs. ENE-negative: stage IIIA, 86.3% vs. 93.4%, *P* = 0.067; stage IIIC, 54.0% vs. 62.3%, *P* = 0.102) (Figure 1). Univariate analysis showed that elevated s-CEA, high histologic grade, pathologic stage, presence of ENE, presence of LVI, presence of PNI, and no adjuvant chemotherapy were associated with poor DFS. In multivariate analysis, pathologic stage, presence of ENE, presence of LVI, presence of PNI, and no adjuvant chemotherapy were significant independent prognostic factors for DFS (Table 2).

**Table 2:** Univariate and multivariate analysis of prognostic factors for disease-free survival (DFS) in patients with node-positive colorectal cancer

Univariate analysis				
Variable		No. of patients	5-year DFS rate	*P* value
Age (years)	<60	897 (46.0)	76.1	0.094
	≥60	1051 (54.0)	72.2	
Sex	Female	795 (40.8)	74.8	0.641
	Male	1153 (59.2)	73.6	
s-CEA	Normal	1552 (79.7)	75.3	0.013
	High	396 (20.3)	69.0	
Histology	WD/MD	1751 (89.9)	74.7	0.045
	PD/Muc/SRC	197 (10.1)	68.6	
Tumor location	Colon	1113 (57.1)	75.7	0.062
	Rectum	835 (42.9)	71.8	
pStage	Stage IIIA	226 (11.6)	92.0	<0.001
	Stage IIIB	1427 (73.3)	74.8	
	Stage IIIC	295 (15.1)	56.9	
Extranodal extension	No	1094 (56.2)	80.1	<0.001
	Yes	854 (43.8)	66.4	
LVI	No	1196 (61.4)	79.6	<0.001
	Positive	752 (38.6)	65.3	
PNI	No	1510 (77.5)	78.3	<0.001
	Positive	402 (20.6)	58.1	
Adjuvant CTx	No	253 (13.0)	62.5	<0.001
	Yes	1695 (87.0)	75.5	

Multivariate analysis				
Variable		Hazard ratio	95% CI	*P* value
s-CEA	Normal	1		0.295
	High	1.122	0.905–1.392	
Histology	WD/MD	1		0.426
	PD/Muc/SRC	1.121	0.847–1.483	
Tumor location	Colon	1		0.196
	Rectum	1.154	0.960–1.387	
pStage	Stage IIIA	1		<0.001
	Stage IIIB	2.813	1.717–4.609	
	Stage IIIC	3.612	2.120–6.155	
Extranodal extension	No	1		<0.001
	Yes	1.507	1.245–1.826	
LVI	Negative	1		<0.001
	Positive	1.490	1.227–1.808	
PNI	No	1		<0.001
	Positive	1.652	1.351–2.020	
Adjuvant CTx	No	1		<0.001
	Yes	0.583	0.457–0.743	

CI: confidence interval.

### Prognostic factors for OS

**Figure 2:**
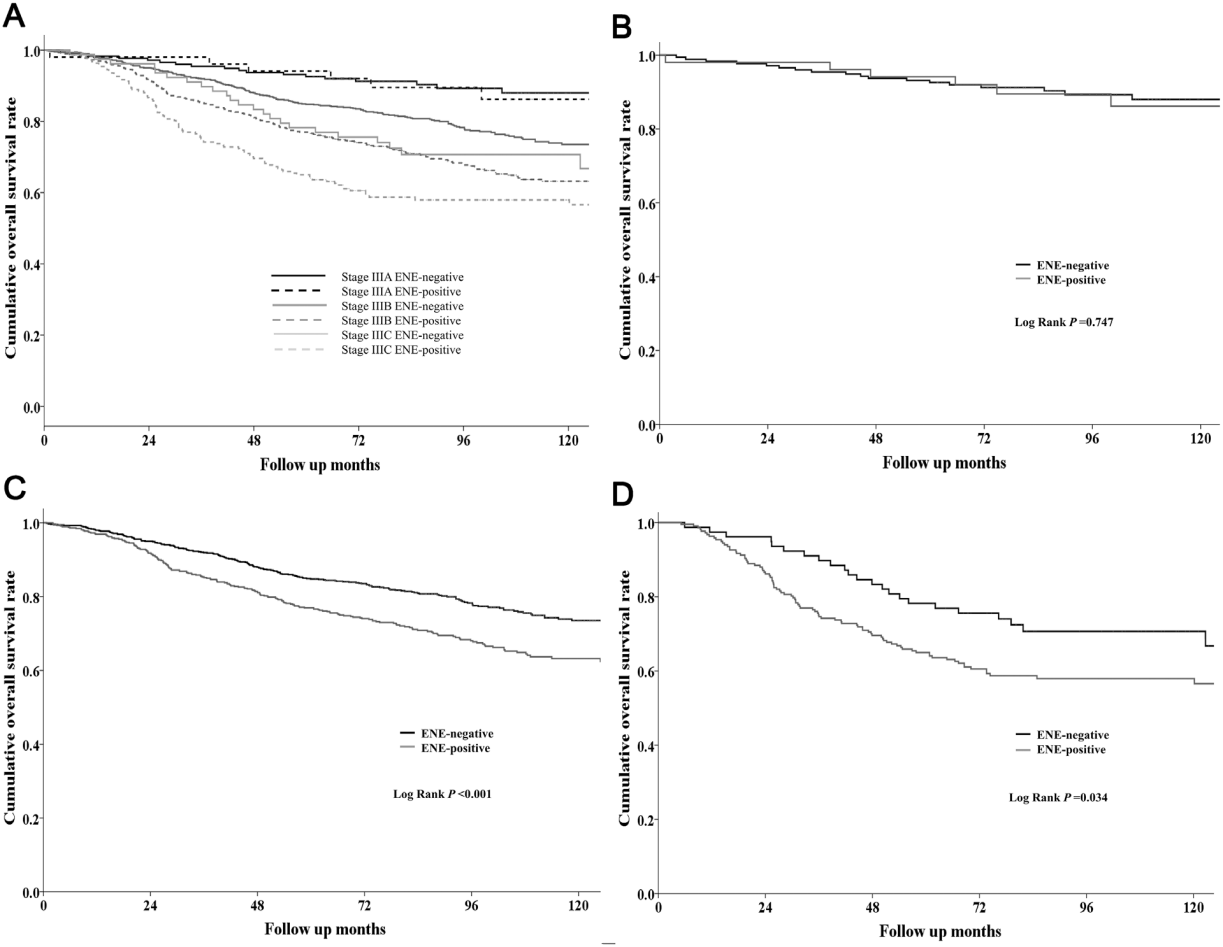
**(A)** Overall survival (OS) rates according to the combination of stage and ENE status of stage III CRC patients. **(B)** OS in stage IIIA patients with (*N* = 51) or without (*N* = 175) ENE. **(C)** OS in stage IIIB patients with (*N* = 586) or without (*N* = 841) ENE. **(D)** OS in stage IIIC patients with (*N* = 217) or without (*N* = 78) ENE.

The 5-year OS rate was lower in patients with ENE-positive tumors than in patients with ENE-negative tumors (74.8% vs. 85.6%, *P* < 0.001). Comparison of OS rates according to stage showed that the 5-year OS was lower in the ENE-positive group than in the ENE-negative group in stage IIIB and stage IIIC (stage IIIB: 76.8% vs. 84.8%, *P* < 0.001; stage IIIC: 64.5% vs. 77.4%, *P* = 0.034), whereas stage IIIA patients showed similar OS (ENE-positive vs. ENE-negative: 92.4% vs. 92.6%, *P* = 0.747) (Figure 2). Univariate analysis showed that old age, elevated s-CEA, high histologic grade, pathologic stage, presence of ENE, presence of LVI, presence of PNI, and no adjuvant chemotherapy were associated with poor OS. In multivariate analysis, old age, pathologic stage, presence of ENE, presence of LVI, presence of PNI, and no adjuvant chemotherapy were significant independent prognostic factors for OS (Table 3).

**Table 3:** Univariate and multivariate analysis of prognostic factors for overall survival (OS) in patients with node-positive colorectal cancer

Univariate analysis				
Variable		No. of patients	5-year OS rate	*P* value
Age (years)	<60	897 (46.0)	85.7	<0.001
	≥60	1051 (54.0)	76.4	
Sex	Female	795 (40.8)	81.5	0.081
	Male	1153 (59.2)	80.5	
CEA	Normal	1552 (79.7)	81.9	0.011
	High	396 (20.3)	76.8	
Histology	WD/MD	1751 (89.9)	82.0	0.020
	PD/Muc/SRC	197 (10.1)	71.1	
Tumor location	Colon	1113 (57.1)	82.1	0.089
	Rectum	835 (42.9)	79.3	
pStage	Stage IIIA	226 (11.6)	92.9	<0.001
	Stage IIIB	1427 (73.3)	81.6	
	Stage IIIC	295 (15.1)	68.0	
Extranodal extension	No	1094 (56.2)	85.6	<0.001
	Yes	854 (43.8)	74.7	
LVI	No	1196 (61.4)	85.7	<0.001
	Positive	752 (38.6)	73.3	
PNI	No	1510 (77.5)	83.4	<0.001
	Positive	402 (20.6)	70.9	
Adjuvant CTx	No	253 (13.0)	57.7	<0.001
	Yes	1695 (87.0)	84.4	

Multivariate analysis				
Variable		Hazard ratio	95% CI	*P* value
Age (years)	<60	1		<0.001
	≥60	1.685	1.390–2.043	
CEA	Normal	1		0.206
	High	1.140	0.930–1.398	
Histology	WD/MD	1		0.091
	PD/Muc/SRC	1.260	0.964–1.646	
pStage	Stage IIIA	1		<0.001
	Stage IIIB	1.906	1.281–2.836	
	Stage IIIC	2.405	1.542–3.753	
Extranodal extension	No	1		<0.001
	Yes	1.527	1.276–1.826	
LVI	Negative	1		<0.001
	Positive	1.502	1.250–1.805	
PNI	No	1		<0.001
	Positive	1.596	1.311–1.942	
Adjuvant CTx	No	1		<0.001
	Yes	0.346	0.283–0.423	

### Comparison of survival according to stage and ENE status

To investigate the prognostic effect of ENE in the different stages, univariate analysis of DFS and OS was performed according to a combination of stage and ENE status. In addition, multivariate analysis was performed after adjusting for age (<60 vs. ≥60), sex, s-CEA (normal vs. high), differentiation (well-differentiated, moderately differentiated vs. poorly differentiated, signet ring cell type, mucinous type), tumor location (colon vs. rectum), LVI status, PNI status, and adjuvant chemotherapy.

In univariate and multivariate analysis, there was no statistically significant difference in DFS and OS rate between ENE-positive stage IIIB tumors and ENE-negative stage IIIC tumors (HR for DFS: 0.964 (0.630-1.473), p=0.865, HR for OS: 0.725 (0.468- 1.123), p=0.15) (Tables 4 and 5). Especially, hazard ratio of ENE-positive stage IIIB to ENE-negative stage IIIC tumors for DFS is close to 1 meaning their risks are no statistically different as well as clinically equivalent.

**Table 4:** Univariate and multivariate analysis (after adjusting for age, sex, s-CEA, differentiation, tumor location, LVI, PNI, and adjuvant chemotherapy) of DFS according to stage and ENE status

Univariate analysis				
Variable	Reference	Hazard ratio	95% CI	*P* value
IIIA_ENE (-)		0.171	0.090–0.323	<0.001
IIIA_ENE (+)		0.404	0.190–0.859	0.019
IIIB_ENE (-)	IIIB_ENE (+)	0.629	0.508–0.779	<0.001
IIIC_ENE (-)		1.111	0.730–1.689	0.624
IIIC_ENE (+)		1.587	1.232–2.044	<0.001

Multivariate analysis				
Parameter	Reference	Hazard ratio	95% CI	*P* value
IIIA_ENE (-)		0.207	0.109–0.394	<0.001
IIIA_ENE (+)		0.467	0.219–0.998	0.049
IIIB_ENE (-)	IIIB_ENE (+)	0.657	0.529–0.815	<0.001
IIIC_ENE (-)		0.964	0.630–1.473	0.865
IIIC_ENE (+)		1.280	0.980–1.672	0.070

**Table 5:** Univariate and multivariate analysis (after adjusting for age, sex, CEA, differentiation, tumor location, LVI, PNI, and adjuvant chemotherapy) of OS according to stage and ENE status

Univariate analysis				
Variable	Reference	Hazard ratio	95% CI	*P* value
IIIA_ENE (-)		0.314	0.198–0.498	<0.001
IIIA_ENE (+)		0.367	0.173–0.780	0.009
IIIB_ENE (-)	IIIB_ENE (+)	0.651	0.532–0.795	<0.001
IIIC_ENE (-)		0.863	0.560–1.330	0.504
IIIC_ENE (+)		1.434	1.116–1.843	0.005

Multivariate analysis				
Parameter	Reference	Hazard ratio	95% CI	*P* value
IIIA_ENE (-)		0.373	0.234–0.594	<0.001
IIIA_ENE (+)		0.436	0.205–0.930	0.032
IIIB_ENE (-)	IIIB_ENE (+)	0.671	0.548–0.822	<0.001
IIIC_ENE (-)		0.725	0.468–1.123	0.150
IIIC_ENE (+)		1.301	1.000–1.692	0.050

## DISCUSSION

In the present study, the ENE rate was 43.8% for all stage III CRCs. Tumor stage was positively related to the incidence of ENE (22.5% in stage IIIA tumors, 41.1% in stage IIIB, and 73.5% in stage IIIC). ENE was also associated with young age, rectal cancer, advanced T and N stages, and LVI/PNI. In a meta-analysis by Veronese et al. [16], the ENE rate was 45.7% for node-positive CRC and ENE was associated with high T stage, high-grade tumors, and advanced tumor stage. The incidence of ENE and the results of our study were consistent with those reported by Veronese et al., strongly suggesting that ENE is closely linked to tumor aggressiveness.

The presence of ENE in metastatic lymph nodes is a negative prognostic factor for cancers of several organs, including stomach [9, 17], esophageal [18], papillary thyroid [10], breast [12], bladder [11], and non-small cell lung [14] cancers. In particular, the presence of ENE in penile cancer [13] and vulvar cancer [15] is also taken into account in the 7^th^ AJCC cancer staging system [2].

The standard definition of ENE is extracapsular growth of tumor cells, invasion of the perinodal fat, or continuous extranodal location of tumor cells. Some studies use alternative definitions. ENE has been defined as an extension of lymph node metastatic cells through the nodal capsule into the perinodal fatty tissue and/or extranodal location of tumor cells [19]. According to this definition, free tumor deposits may be included in the ENE-positive category. Therefore, we used the standard definition of ENE. However, in the above meta-analysis, stratification of patients according to the definition of ENE (classical vs. alternative definition) did not significantly change the survival outcomes [16].

The prognostic implication of ENE in CRC was investigated previously, with most studies reporting that ENE-positive status is associated with poor prognosis in CRC [16, 19–23]. In the present study, Kaplan-Meier survival curves showed significant differences in both DFS and OS between ENE-positive and ENE-negative patients. Subgroup analysis indicated that the difference was more significant in stage IIIB cancers. However, there was a tendency towards different survival outcomes between ENE-positive and ENE-negative patients in stage IIIA and IIIC cancers. This result can be attributed to the relatively good prognosis of stage IIIA cancers and the small sample size for stage IIIC cancers. Multivariate analysis also showed that ENE-positive status was a statistically significant independent prognostic factor for poor DFS and OS.

In the multivariate analysis, stage, presence of LVI, presence of PNI, and no adjuvant chemotherapy were independent prognostic factors for poor DFS and OS. Pathologic stage is the most powerful prognostic factor [2, 24]. The presence of LVI or PNI is considered a negative prognostic indicator according to previous studies [25–28]. It has been recognized since 1990 that adjuvant chemotherapy significantly improves the OS and DFS outcomes of stage III colorectal cancer patients [29–31]. Similarly, in the present study, multivariate analysis showed that lack of adjuvant chemotherapy was an independent prognostic factor for poor DFS and OS.

Some authors wonder that ENE is important for both colon and rectal cancers [32, 33]. We analyzed the DFS for both colon and rectal cancers separately. 5-year DFS rate was significantly lower in the patients with ENE both colon and rectal cancers, respectively (ENE-negative vs. ENE-positive; 81.2% vs. 68.0%, *P*<0.001) (ENE-negative vs. ENE-positive; 78.3% vs. 64.9%, *P*<0.001) (Supplementary Figure 1). ENE may be an important prognostic factor for both colon and rectal cancers. The invasion by tumor cells of perinodal adipose tissue, indeed, is the same mechanism in both these locations that permits to the tumor to increase aggressiveness and metastatic potential. We will consider investigating the prognostic impact of ENE on colon and rectal cancer separately in our future study.

Taking the ENE status into consideration, DFS and OS were compared between stage IIIB and stage IIIC cancers. There were no statistically significant differences in DFS and OS between ENE-positive stage IIIB tumors and ENE-negative stage IIIC tumors. This suggests that the ENE status affects the staging system. Furthermore, the presence of ENE was more than 40% in stage III CRCs. This is another reason for which ENE should be considered by future staging system. The presence of ENE is not a rare condition. In line with the AJCC 7^th^ staging system, it is important to look for free tumor deposits in the subserosa, mesentery, or non-peritonealized pericolic or perirectal tissue [2, 34] in CRC and to assess ENE status in penile and vulvar cancer. The present findings suggest that ENE status in stage III CRC needs to be considered as an important factor in the staging system. In 2017, the AJCC 8^th^ edition [35] was published. However, the 8^th^ edition of AJCC staging manual also does not consider ENE status.

In conclusion, the presence of ENE was closely related to tumor aggressiveness in CRC. ENE was a significant independent prognostic factor for DFS and OS after curative resection for stage III CRC. ENE assessment should be included in histopathological evaluations and ENE status should be considered as part of the staging system for this disease.

## PATIENTS AND METHODS

### Patients

The medical records and database of 1,948 CRC patients who underwent surgery at Asan Medical Center, Seoul, Korea, between January, 2003, and December, 2010, were retrospectively reviewed. All patients included in this study met the following criteria: (1) a diagnosis of stage III CRC, (2) histologically proven adenocarcinoma, and (3) history of curative resection (R0). Patients who received preoperative neoadjuvant therapy, those with hereditary CRC (familial adenomatous polyposis and hereditary nonpolyposis colorectal cancer) and those with multiple colorectal cancers were excluded.

### Evaluation

Before surgery, all patients underwent a staging workup, including colonofiberscopy, chest radiography, computed tomography (CT) of the abdomen and pelvis, and measurement of serum carcinoembryonic antigen (s-CEA) levels. In some patients, positron emission tomography (PET) scan, single contrast-enhanced magnetic resonance imaging (MRI) of the liver, and/or chest CT scan were performed to further characterize the lesion’s risk of malignancy. Serum CEA was measured by enzyme immunoassay (ELISA-2-CEA kit®; CIS Bio International, Marcoule, France). The normal s-CEA concentration was set at ≤6 ng/mL. Tumors were pathologically staged in accordance with the cancer staging manual (AJCC 7^th^ edition).

### Histologic evaluation

**Figure 3:**
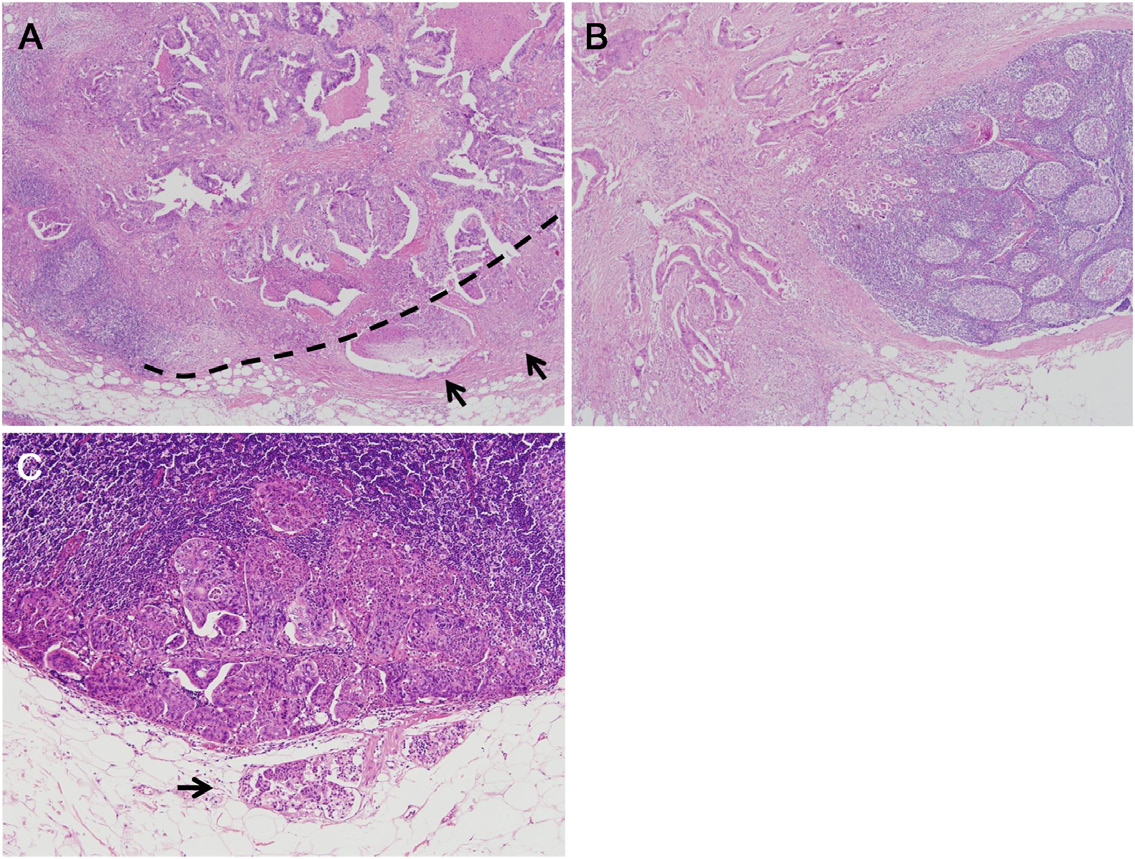
Representative examples of lymph node metastasis patterns **(A)** Extranodal extension (ENE) positive: tumor cells invading fat tissue (arrows) beyond the boundary of the lymph node (dashed line) (×4 objective lens). **(B)** Extranodal extension negative: tumor cells present outside the lymph node but continuous with the primary tumor (×4 objective lens). **(C)** Extranodal extension negative: tumor cells present outside the lymph node but confined to endolymphatic spaces (arrow) (×10 objective lens).

The ENE status of all specimens was examined by two pathologists, including a fellow and a faculty pathologist, and the final diagnosis was made on the basis of intradepartmental consultation with staff specialized in CRC. ENE was defined as cancer cells infiltrating the extranodal adipose tissue beyond the capsule of the lymph node (Figure 3A). Tumor cells that were present outside the lymph node and continuous with the primary tumor (Figure 3B) or confined to endolymphatic spaces (Figure 3C) were not considered ENE. In addition, tumor deposits, and clear lymphovascular or perineural invasion were not considered ENE. When lymphovascular invasion was equivocal, elastic staining, or immunohistochemistry for CD31 or D2-40 was generously performed. The tumor was considered ENE-positive when one or more of the metastatic lymph nodes showed ENE.

### Adjuvant chemotherapy

Of the 1,984 patients in the study cohort, 1,780 (91.4%) received postoperative chemotherapy as follows: 329 patients (16.9%) received 5-fluorouracil; 821 (42.1%) received capecitabine; 416 (21.4%) received oxaliplatin; 96 (4.9%) received an oral pyrimidine analogue or oral 5-fluorouracil; and 118 (6.1%) patients received chemotherapy at another hospital. The remaining 168 patients (8.6%) did not receive adjuvant chemotherapy. For data analysis, patients who received either 5-fluorouracil-based chemotherapy intravenously or capecitabine-/oxaliplatin-based chemotherapy during the determined periods were considered as having complete adjuvant chemotherapy. Of the 835 rectal cancer patients, 330 (39.5%) received postoperative radiotherapy.

### Follow-up

Patients underwent a standardized postoperative follow-up (including clinical examinations, complete blood counts, blood chemistry tests, measurements of s-CEA levels, and chest radiography) every 3 months for the first 2 postoperative years and every 6 months thereafter. Patients also underwent abdominal and pelvic CT scan every 6 months. Colonoscopy was performed within 1 year of surgery and then once every 2–3 years. If recurrence was suspected, patients underwent CT scan, MRI, and/or PET scan. Recurrence was diagnosed pathologically (by surgical resection or biopsy) and/or radiologically.

### Statistical analysis

Categorical variables were compared using chi-squared tests, and continuous variables were compared using independent sample *t*-tests. The Kaplan-Meier method was used to compare disease-free survival (DFS) and overall survival (OS) rates. Univariate and multivariate analyses of factors associated with DFS rates were performed using Cox proportional hazards (PH) regression analyses to estimate the hazard ratios and yield 95% confidence intervals (CIs). All statistical tests were two-sided, and *P* < 0.05 was considered statistically significant. Statistical analyses were performed using SPSS 21.0 for Windows (SPSS, Inc., Chicago, IL).

## SUPPLEMENTARY MATERIALS FIGURE


