# Supplementary material for: Extranodal extension status is a powerful prognostic factor in stage III colorectal cancer
Source: Oncotarget. 2017 May 26;8(37):61393–403. doi: 10.18632/oncotarget.18223 (PMC5617432; doi:10.18632/oncotarget.18223)
Supplement: Supplementary file 1 [file oncotarget-08-61393-s001.pdf]

Extranodal extension status is a powerful prognostic factor in stage III colorectal cancer

SUPPLEMENTARY INFORMATION

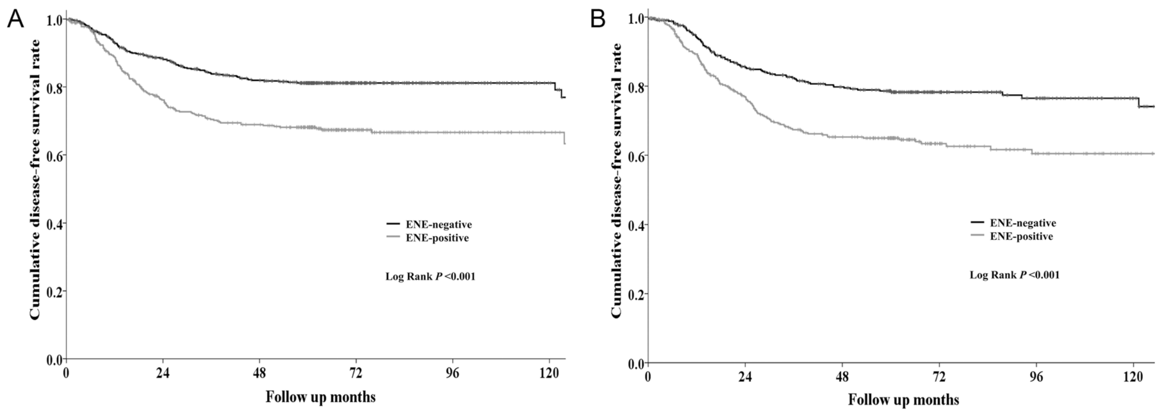

Supplementary Figure 1: (A) DFS rates according to ENE status in colon cancer patients (N=1113). **(B)** DFS rates according to ENE status in rectal cancer patients (*N*=835).
